# The current status of health care indices and functional independence among older adults: data from HelpAge international-jordan study

**DOI:** 10.1007/s40520-024-02738-2

**Published:** 2024-05-30

**Authors:** Mohammad Abufaraj, Lana Alhalaseh, Mohammed Q. Al-sabbagh, Zaid Eyadat, Walid Al Khatib, Osama A Samara, Immanuel Azaad Moonesar, Lee Smith, Raeda Al qutob

**Affiliations:** 1 Division of Urology, Department of Special Surgery, Jordan University Hospital, The University of Jordan, Amman, 11942 Jordan; 2 Center of strategic studies, The University of Jordan, Amman, Jordan; 3 Division of Geriatrics, Department of Family and Community Medicine, School of Medicine, The University of Jordan, Amman, Jordan; 4 School of Medicine, the University of Jordan, Amman, Jordan; 5 Department of Radiology and nuclear medicine, University Hospital, The University of Jordan, Amman, Jordan; 6 Health Administration & Policy, Mohammed Bin Rashid School of Government, Dubai, United Arab Emirates; 7 Centre for Health, Performance and Wellbeing, Anglia Ruskin University, Cambridge, UK; 8 Department of Family and Community Medicine, School of medicine, The University of Jordan, Amman, Jordan

**Keywords:** Delivery of health care, Chronic disease, Multimorbidity, Jordan, Functional dependence

## Abstract

**Background:**

Health services should anticipate the changing pattern of illnesses associated with population aging to promote healthy aging.

**Aim:**

We aimed to evaluate health indices and chronic illnesses and their relationship with functional independence in community Syrian refugees & Jordanian elderly.

**Methods:**

A stratified sample of 1,718 community older adults aged ≥ 60-year-old from four major Jordanian governorates was interviewed in this cross-sectional study. Katz Index of Independence in Activities of Daily Living was utilized to assess functional status. Data were analyzed using STATA 15.

**Results:**

Despite the similarities in baseline function, Syrian refugees had more multimorbidities but less active health insurance, accessibility to healthcare services and availability of medications and medical devices than Jordanians. Two-thirds had multimorbidities; with heart diseases, musculoskeletal conditions, hypertension, and diabetes being the most commonly reported chronic illnesses. Females had significantly more multimorbidities, and functional dependence, yet less education, income and accessibility to healthcare services. The mean Katz Index score was 4.99 ± 1.61. Significant predictors of functional dependence included increasing age, lower level of education, and some chronic illnesses.

**Conclusion:**

National inclusive plans to support vulnerable older adults especially refugees and older women, provide health insurance, enhance access to health care facilities, and manage chronic medical illnesses comprehensively are urgently needed to improve independence of community-living older adults and to promote healthy aging.

**Supplementary Information:**

The online version contains supplementary material available at 10.1007/s40520-024-02738-2.

## Introduction

The global population is rapidly aging in both developing and developed countries due to improvements in healthcare, reduction in mortality rates, urbanization and a subsequent demographic transition [1]. With aging, the prevalence of chronic illnesses and multimorbidity is increasing in parallel with rising social and healthcare demands and expenditures on one hand and decreasing functional independence and survival on the other hand, which may become an important public policy problem [2–5].

The most important consideration for an older person is likely to be their functionality rather than the presence or absence of diseases [2]. The World Health Organization, therefore, has advocated for “Healthy aging” which is “the process of developing and maintaining the functional ability that enables wellbeing in older age” [6]. Functional ability is measured by the capacity to meet basic life demands and perform needed life roles that include Basic and Instrumental Activities of Daily Living (ADLs and IADLs; respectively) [6]. It is negatively affected by the accumulation of health deficits and chronic illnesses that are translated as multimorbidities [3].

The Hashemite Kingdom of Jordan is an upper-middle-income country with a population of 11.1 million and an estimated life expectancy at birth of 73.3 years [7, 8]. It hosts the second-highest share of refugees per capita in the world where more than 1.3 million Syrian refugees have fled the war and are registered with the United Nations High Commissioner for Refugees (UNHCR) which works closely with the Government of Jordan and other partners, like HelpAge International, to provide protection and assistance to refugees and Jordanians affected by the refugee influx [9]. They aim to improve the lives of older women and men in low- and middle-income countries, especially those in crisis-prone areas by responding to humanitarian crises, advancing gender equality and advocating for the rights of older people through challenging ageism.

Due to the refugees’ influx, the development of health policies in Jordan has been challenging, especially for this vulnerable group of older adults which lacks the necessary data that help frame such inclusive policies. Previous research in Jordan highlighted the need for high-quality, nationwide studies to address the patterns of chronic illnesses, functional status and health indices among older adults in order to have customized policies in place [7].

Therefore, this study aimed to explore health indices including the patterns of chronic illnesses and multimorbidity and their relationship with ADLs in community older adults, including Syrian refugees and Jordanians living in northern Jordan, with particular emphasis on gender differences in an attempt to help phrase healthcare strategies tailored to these specific needs.

## Methods

### Study design

This is a cross-sectional survey-based study conducted by the Center for Strategic Studies at the University of Jordan in collaboration with HelpAge International-Jordan. Using a two-stage stratified-cluster sampling design, 15 field supervisors and 45 data collectors interviewed 1718 participants representing Jordanian and Syrian adults from both genders, aged ≥ 60 years. The samples were captured from the four major governorates (Amman, Zarqa, Irbid and Mafraq) hosting Syrian refugees, excluding those residing in refugees’ camps.

The latest Jordan Population and Housing Census 2015 was used as a sampling frame for Jordanians.

A sample of 860 households was randomly drawn to represent the Jordanian population in the four targeted governorates. It was designed in a probability proportional to size (PPS) way to provide valid and reliable survey estimates across the four governorates - rural and urban areas. Furthermore, each governorate was subdivided into area units called census blocks, which were the Primary Sampling Units (PSU-Blocks) for this survey (on average, a PSU comprises 50–70 households). The PSU-Blocks were then regrouped to form clusters. From each PSU, eight households were randomly drawn with an equal probability systematic selection. A household was defined as a group of people living in the same dwelling space who eat meals together, acknowledging the authority of a man or a woman as the household head. After the household selection and obtaining the permission of household residents to participate in the survey, all the eligible household members (has to be over 60 years old) were entered into the CSPRO program, which ran a random selection to choose one member from each household.

The sample size from each governorate was proportionate to the population’s size (see the Supplementary Table S1).

To update the sampling frame for Syrians, a pre-listing of households took place, where Syrian households were visited and entered into a listing frame (the same listing questionnaire was used). This sampling frame enabled weight calculation and the selection of the final Syrian sample. This was followed by determining the sampling blocks (PSU), which were 108, then 8 households were randomly selected from each block to fulfill the pre-calculated sample size of 858.

### Measurement instrument

The study instrument was designed by HelpAge International, supported by a steering committee that included statisticians, geriatric consultants, and other experts in the field. Survey items included sociodemographic variables: age, gender, marital status, monthly income in Jordanian Dinar (JOD, 1 JOD = 1.41 USD), Nationality (Jordanian or Syrian), region of residence (Amman, Al-Zarqa, Irbid, and Mafraq), and the level of education (Illiterate, less than secondary, and secondary and/or higher education). The interviewers asked about age-related health indices: living alone, needing assistance in their daily life, having chronic medical illnesses (self-report), having active health insurance, accessibility to healthcare services, access to medications, and availability of blood pressure or blood sugar measuring devices in patients with hypertension and diabetes, respectively.

Chronic illnesses are then grouped into major categories that included: heart diseases (including heart failure, angina and valvular heart disease), hypertension, dyslipidemia, lung diseases (including asthma, bronchitis and interstitial lung diseases), gastrointestinal and liver diseases, diabetes mellitus, thyroid diseases, musculoskeletal conditions (including arthritides and back pain), neurological diseases (including stroke, headaches, and seizures), kidney (including urinary tract) diseases, neoplasms and cancers, hematological diseases, and ophthalmic and dermatological diseases (requiring repeated prescriptions). Multimorbidity is defined as having at least two concurrent chronic illnesses [10].

Katz Index of Independence in Activities of Daily Living (ADLs) was utilized to evaluate the functional status of the participants [11]. It assesses six basic ADLs (Bathing, Dressing, Toileting, Transferring, Continence, and Feeding) with two-option responses for each item (dependent = 0, independent = 1), yielding a total score of 6. The higher the score, the higher the independence in ADLs.

### Data analysis

Data were analyzed using STATA (Stata Statistical Software: Release 16. College Station, TX: StataCorp LLC). First, gender differences in demographic variables, healthcare indices and chronic illnesses were evaluated using chi-square test. Then, women and men were stratified into two groups based on their perception of needing assistance in their daily life, and differences between each stratum were assessed using chi-square test. Statistically significant results were regarded if the *p*-value was < 0.05.

A linear regression analysis was used to assess the impact of sociodemographic variables and chronic illnesses on the Katz Index of Independence in ADLs score. Variables were first evaluated using univariate linear regression analysis, and the variables that were significant to the outcome were included in the final multivariable linear regression model to estimate the beta coefficient (β) and 95% confidence interval (CI).

## Results

### Sample characteristics

Overall, 1,718 participants completed the interview. The sample was equally distributed between Jordanians (50.1%) and Syrian refugees (49.9%) and between men and women. Table 1 shows that one thousand (58.2%) participants were between 60 and 70 years old, living in the capital Amman (39.1%), married (61.6%), had secondary education or less (44.1%), and reported a household income of ≤ 200 JOD (66.0%). The mean Katz Index score was 4.99 ± 1.61.

Independent sample t-test showed that participants who declared their need for assistance with their daily life (*n* = 493) had significantly lower mean Katz Index scores (3.86 ± 1.98) compared to those who did not perceive a need for assistance (5.44 ± 1.16), *P* < 0.001.

There were significant gender differences in reported and measured (Katz Index) functional dependence, marital status, educational level, the status of living alone and household income (*P* < 0.05).

**Table 1:** Socioeconomic characteristics of Jordanian & Syrian older adults living in Jordan

Variable		Total	Men (%)	Dependent Men^a^	P value	Women (%)	Dependent women^a^	P value	Gender difference (P value)
Total numbers		1,718 (100.0)	862 (50.2)	180 (20.9)		856 (49.8)	313 (36.6)		**< 0.005**
Katz Index of ADLs (Mean ± SD)		4.99 ± 1.61	5.12 ± 1.54	3.58 ± 1.99	**< 0.005**	4.86 ± 1.67	4.03 ± 1.96	**< 0.005**	**0.001**
Age group (year)	60–69	1,000 (58.2)	493 (49.3)	73 (14.8)	**< 0.01**	507 (50.7)	135 (26.6)	**< 0.01**	0.4
	70–79	509 (29.6)	254 (49.9)	58 (22.8)		255 (50.1)	114 (44.7)		
	80–89	176 (10.2)	96 (54.6)	36 (37.5)		80 (45.5)	52 (65.0)		
	90–99	31 (1.8)	17 (54.8)	12 (70.6)		14 (45.2)	12 (85.7)		
	≥ 100	2 (0.1)	2 (100.0)	1 (50.0)		0 (0)			
Region	Capital (Amman)	671 (39.1)	337 (50.2)	62 (18.4)	**< 0.01**	334 (49.8)	126 (37.7)	0.63	1.0
	Al-Zarqa	383 (22.3)	193 (50.4)	42 (21.8)		193 (49.6)	62 (32.6)		
	Irbid	432 (25.2)	216 (50.0)	37 (17.1)		216 (50.0)	80 (37.0)		
	Mafraq	232 (13.5)	116 (50)	39 (33.6)		116 (50)	45 (38.8)		
Nationality	Jordanian	860 (50.1)	432 (50.2)	85 (19.7)	0.38	428 (49.8)	174 (40.7)	**0.013**	0.96
	Syrian	858 (49.9)	430 (50.1)	95 (22.1)		428 (49.9)	139 (32.5)		
Marital status	Unmarried	71 (4.1)	18 (25.4)	4 (22.2)	**0.01**	53 (74.7)	13 (24.5)	**0.007**	**< 0.005**
	Married	1058 (61.6)	765 (72.3)	149 (19.5)		293 (27.7)	93 (31.7)		
	Widowed	589 (34.3)	79 (13.4)	180 (20.9)		510 (86.6)	207 (40.6)		
Level of education	Illiterate	714 (41.6)	187 (26.2)	66 (35.3)	**< 0.01**	527 (73.8)	210 (39.9)	**0.035**	**< 0.01**
	Less than secondary	757 (44.1)	485 (64.1)	94 (19.4)		272 (35.9)	87 (32.0)		
	Secondary or higher	247 (14.4)	190 (76.9)	20 (10.5)		57 (23.1)	16 (28.1)		
Living alone	Yes	150 (8.7)	40 (26.7)	13 (32.5)	**0.05**	110 (73.3)	53 (48.2)	**0.007**	**< 0.01**
Household income (JOD)	≤ 200	1134 (66.0)	468 (41.3)	126 (26.9)	**< 0.01**	666 (58.7)	247 (37.1)	0.43	**< 0.01**
	201–300	317 (18.5)	197 (62.2)	30 (15.2)		120 (37.9)	45 (37.5)		
	301–499	215 (12.5)	156 (72.6)	21 (13.5)		59 (27.4)	16 (27.1)		
	≥ 500	52 (3.0)	41 (78.9)	3 (7.3)		11 (21.2)	5 (45.5)		

^a^ Dependence is determined by the answer to the question (Do you need help performing any of your ADLs?)

### Gender differences in healthcare among jordanians & Syrian refugees

Table 2 shows that women from both Jordanian and Syrian nationalities had significantly more multimorbidity (*P* < 0.001), and higher need for chronic medications (*P* < 0.001) compared to men. Nonetheless, they had less access to healthcare than men. Syrian refugees, of both genders, had significantly less favorable healthcare indices compared to their Jordanian counterparts. These include less healthcare insurance (14.3% vs. 85.7%, *P* < 0.001), less access to healthcare (36.3% vs. 63.7%, *P* < 0.001), more multimorbidity (51.2% vs. 48.1%, *P* = 0.027) and less availability of medications for chronic illnesses (39.3% vs. 60.7%, *P* < 0.001) as well as the devices to monitor their diabetes and hypertension (*P* < 0.001).

**Table 2:** Health care Indicators of 1718 Jordanian and Syrian older adults by Gender ^a^

		Jordanians			Syrians			
Health care indicators	Total (%)	Men 432 (%)	Women 428 (%)	P-value	Men 430 (%)	Women 428 (%)	P-value	Nationality P-value
Active health insurance	901 (52.4)	389 (90.0)	383 (89.5)	0.822	60 (14.0)	69 (16.1)	0.391	**< 0.001**
Accessible healthcare facility	842 (49.0)	288 (66.7)	248 (57.9)	**0.009**	176 (40.9)	130 (30.4)	**0.001**	**< 0.001**
Needing regular medications for at least one chronic illness	1213 (70.6)	257 (59.5)	336 (78.5)	**< 0.001**	277 (64.4)	343 (80.1)	**< 0.001**	0.138
Availability of chronic illnesses’ medications ^**b**^	792 (65.3)	210 (81.7)	271 (80.7)	0.832	135 (48.7)	176 (51.3)	0.572	**< 0.001**
Multimorbidity ^**c**^	1105 (64.3)	234 (52.4)	297 (69.4)	**< 0.001**	256 (59.5)	318 (74.3)	**< 0.001**	**0.027**
Availability of blood sugar monitor in patients with diabetes	339 (49.6)	97 (59.1)	113 (60.8)	0.827	61 (42.1)	68 (36.0)	0.260	**< 0.001**
Availability of blood pressure monitor in patients with hypertension	322 (34.7)	92 (46.2)	113 (43.6)	0.636	61 (29.8)	56 (21.2)	**0.041**	**< 0.001**

^**a**^ Percentages are within Gender category

^**b**^ Out of those needing regular medications

^**c**^ Multimorbidity is defined as having two or more concurrent chronic illnesses

Table 3 shows the prevalence of chronic illnesses among the participants. Hypertension was the most common chronic illness reported by participants while neoplasms were the least common. Male sex was significantly associated with heart disease, whereas female sex was more significantly associated with hypertension, diabetes, thyroid diseases and musculoskeletal conditions.

**Table 3:** Prevalence and gender differences of chronic diseases in 1718 adults ≥ 60 years old

Chronic Diseases Category	Total (%)	Males (%)	Dependent Males (%)	P value	Females (%)	Dependent Females (%)	P value	Gender difference (P value)
Heart diseases	421(24.5)	233(55.3)	72 (30.9)	**< 0.01**	188(44.7)	97 (51.6)	**< 0.01**	**0.015**
Hypertension	927 (53.9)	404(43.6)	100 (20.9)	**0.01**	523(56.4)	218 (41.7)	**< 0.01**	**< 0.005**
Pulmonary diseases	139 (8.1)	70 (50.4)	20 (28.6)	0.1	69 (49.6)	33 (47.8)	**0.04**	0.96
Dyslipidemia	246 (14.3)	113 (45.9)	40 (35.4)	**< 0.01**	133 (54.1)	59 (44.4)	**0.04**	0.15
Gastrointestinal diseases	150 (8.7)	65 (43.3)	15 (23.1)	0.65	85 (56.7)	32 (37.7)	0.827	0.079
Diabetes Mellitus	684 (39.8)	309 (45.2)	83 (26.9)	**0.001**	375 (54.8)	146 (38.9)	0.20	**0.001**
Thyroid diseases	70 (4.1)	13 (18.6)	3 (23.1)	0.84	57 (81.4)	26 (45.6)	0.142	**< 0.005**
Musculoskeletal conditions	813 (47.3)	319 (39.2)	91 (28.5)	**< 0.01**	494 (60.8)	200 (40.5)	**< 0.005**	**< 0.005**
Dermatological diseases	33 (1.9)	13 (39.4)	9 (69.2)	**< 0.01**	20 (60.6)	14 (70.0)	**0.002**	0.21
Neurological diseases	191 (11.1)	91 (47.6)	37 (40.7)	**< 0.01**	100 (52.4)	50 (50.0)	**0.002**	0.46
Kidney & Urological diseases	153 (8.9)	80 (52.3)	27 (33.8)	**0.003**	73 (47.7)	34 (46.6)	**0.05**	0.58
Neoplasms	29 (1.7)	14 (48.3)	4 (28.6)	0.475	15 (51.7)	9 (60.0)	**0.05**	0.84
Hematological diseases	35 (2.0)	13 (37.1)	3 (23.1)	0.84	22 (62.9)	10 (45.5)	0.38	0.12
Ophthalmic diseases	384 (22.3)	184 (47.9)	52 (28.3)	**0.005**	200 (52.1)	84 (42.0)	0.068	0.315

### Predictors of independence in ADL

Overall, 649 participants (37.75%) reported being dependent in at least one ADL, with transferring (24.33%) and bathing (23.75%) having the highest dependency rates (Supplementary Table-2). Table 4 shows the Multivariable-adjusted linear regression analysis evaluating the impact of different factors on Independence in Activities of Daily Living measured by Katz Index. Increasing level of education was a positive predictor of higher ADLs, with a dose-response relationship (“Less than secondary” vs. “Illiterate”) and (“secondary & higher” vs. “Illiterate”). Increasing age was significantly associated with dependence in ADLs, so were the following chronic illnesses: heart diseases, diabetes, musculoskeletal conditions, neurological diseases and kidney diseases. Gender was not found a significant predictor of dependence in ADLs.

**Table 4:** Multivariable-adjusted linear regression analysis evaluating the impact of different factors on Independence in Activities of Daily Living measured by Katz Index

Variable		β	95% CI^a^	P value
Age (Years)		-0.94	-1.10 – -0.78	**<0.01**
Gender	Males (ref)			
	Females	0.07	-0.26 – 0.40	0.67
Marital status	Unmarried (ref)			
	Married	0.59	-0.07 – 1.24	0.08
	Widowed	0.20	-0.44 – 0.84	0.54
Level of Education	Illiterate (ref)			
	Less than secondary	0.52	0.26 – 0.78	**<0.01**
	Above secondary	1.23	0.74 – 1.72	**<0.01**
Chronic illnesses	Others (ref)			
	Heart diseases	-0.76	-1.02 – -0.51	**<0.01**
	Diabetes	-0.57	-0.91 – -0.33	**<0.01**
	Musculoskeletal	-0.52	-0.77 – -0.28	**<0.01**
	Neurological diseases	-1.03	-1.36 – -0.70	**<0.01**
	Kidney diseases	-0.71	-1.08 – -0.35	**<0.01**

^a^ CI: Confidence interval

## Discussion

In this representative sample of older adults living in northern Jordan, Syrian refugees had less favorable healthcare indices than their Jordanian counterparts despite the similarities in their baseline function. Two-thirds had multimorbidities; with heart diseases, musculoskeletal conditions, hypertension, and diabetes being the most commonly reported chronic illnesses. Female gender was significantly associated with more multimorbidities and functional dependence, yet less education, income and accessibility to healthcare services. Significant predictors of functional dependence included increasing age, level of education, and some chronic illnesses.

Our results showed that 37% of the participants had a limitation in at least one ADL as measured by the Katz Index score, which is higher than the reported prevalence in some studies in China (26.6%) [12], Malaysia (14%) [13] and across the European Union where prevalence rates ranged from 8 to 28% [14]. On the other hand, similar rates of functional dependence were seen in Spain [14], and higher rates were seen in India [15]. Alhalaseh showed that less than 10% of Jordanian older adults were dependent in at least one ADL, yet when Instrumental Activities of Daily Living (IADLs) were included, 33.8% were dependent in at least one IADL/ADL [16]. These high rates of dependence in ADLs might be related to many factors; firstly, more than 64% of the study participants had multimorbidity which is much higher than the reported global (51.0%) [4] and previous national numbers (32.9%) [16] in samples representing the same age groups. Multimorbidity is known to be associated with functional decline [4]. Secondly, compared to the previous study done in Jordan, this cohort had lower levels of education and less monthly income. In fact, half of the cohort is comprised of refugees, of which 85% had less than a secondary level of education. Research findings reiterate the higher rates of multimorbidity in groups with fewer educational qualifications and lower household income [4, 5, 17, 18]. It is argued that higher levels of education are associated with the adoption of healthier lifestyles that help maintain functional status [19] and are linked with the development of many brain circuits that protect cognitive and functional abilities in the elderly [19–21].

To complicate things more, it was found that Syrian refugees had significantly less active health care insurance and accessibility to health services compared to Jordanians. In comparison, the overall national health insurance coverage in Jordan is 86% [22].

Therefore, screening programs targeting populations with lower socioeconomic status and educational levels are essential anticipatory proactive measures that may help minimize dependence in ADLs. This necessitates providing active health insurance and improving accessibility to primary care which is the foundation of successful screening programs. Additionally, focusing on the wider context of multimorbidity, rather than single diseases, as most clinical practice guidelines recommend, leads to a better understanding of the impact of chronic diseases on older adults from a patient-centered perspective [7]. Advocating for geriatric training of primary care providers is expected to enhance their understanding of multimorbidity as a geriatric concept.

Not unexpectedly, it was found that women had a significantly higher prevalence of functional dependence and multimorbidity but less accessibility to healthcare. This gender preponderance is corroborated by research studies, and it indicates an association that could be related to biological, sociocultural, environmental, or economic factors [4, 18, 23]. In our cohort, women were significantly more illiterate than men. They also had a significantly lower monthly income and lived alone more frequently than men. Targeting these vulnerable older women and improving their accessibility to healthcare is of paramount priority to help manage their multimorbidity, hence improving functional dependence.

Interestingly, three out of the four most commonly reported chronic illnesses in our sample were significant predictors of lower Katz Index of Independence in ADLs score, namely: heart diseases, musculoskeletal conditions, and diabetes. These results highlight one of the major challenges facing healthcare in the aging populations where the focus of healthcare practices should shift from managing diseases and pathologies to managing patients to encourage independence and healthy aging [3]. Moreover, this indicates a pressing need for further research investigating the interplay of chronic diseases, multimorbidities and functional independence among vulnerable older adults to facilitate framing national guidelines and policies that are inclusive [7].

## Conclusion

While more than two-thirds of the study participants were independent in ADLs, the results showed that older adults have a high level of multimorbidities and functional dependence and low accessibility to healthcare and health insurance coverage, especially among older adults’ refugees. Female gender was significantly associated with more multimorbidities, and functional dependence, yet less education, income and accessibility to healthcare services.

Healthcare providers and policymakers should understand the predictors of poor functional status especially among the vulnerable groups of older women and refugees. Geriatric training should also be advocated to improve the awareness of healthcare providers about the complex needs of older adults. For national plans to be inclusive, they should prioritize health insurance, enhance access to healthcare facilities, and manage chronic medical illnesses comprehensively especially among vulnerable older adults’ refugees and women.

### Limitations of this study

Our study has several limitations worth mentioning. Firstly, its cross-sectional nature makes it unsuitable for drawing cause-effect relationships. Secondly, relying on the self-reported prevalence of diseases and symptoms might be less accurate than using medical records as it is subjected to recall and social desirability biases. Thirdly, mental health issues that include depression and other mood and memory disorders were not enquired about which negatively affects the prevalence of multimorbidity and chronic illnesses and overlooks an integral pillar in functional dependence.

Finally, the association reported in this study may be influenced by residual and unmeasured confounding, which we could not account for. The major strength of our study is, however, the representative sampling technique and the validated, culturally adopted data collection instrument. Moreover, the inclusion of Syrian refugees residing in Jordan, and outside refugee camps, gives contemporary insight into the status of health indices of vulnerable elderly people living currently in Jordan.

### Electronic supplementary material

Below is the link to the electronic supplementary material.

Supplementary Material 1

## Data Availability

The data that support the findings of this study are available from The Center for Strategic Studies. Restrictions apply to the availability of these data, which were used under license for this study. Data are available from the corresponding author upon reasonable request and with the permission of The Center for Strategic Studies.
