# Supplementary material for: The current status of health care indices and functional independence among older adults: data from HelpAge international-jordan study
Source: Aging Clin Exp Res. 2024 May 30;36(1):124. doi: 10.1007/s40520-024-02738-2 (PMC11136843; doi:10.1007/s40520-024-02738-2)
Supplement: Supplementary file 1 — Supplementary Material 1 [file 40520_2024_2738_MOESM1_ESM.docx]

**The Current Status of Health Care Indices and Functional independence in the Older People: Data from HelpAge Study in Jordan**

**Authors:**

Mohammad Abufaraj MD ^1,2^, Lana Alhalaseh MD ^3^, Mohammed Q. Al-sabbagh MD ^4^, Zaid Eyadat PhD ^2^, Walid Al Khatib PhD^2^, Salam Daradkeh MD^5^, Lee Smith PhD^6^, Ra’eda Al-Qutob MD ^7^

**Affiliations:**

^1^ Division of Urology, Department of Special Surgery, Jordan University Hospital, The University of Jordan, Amman, Jordan.

^2^ Center of Strategic Studies, the University of Jordan, Amman, Jordan

^3^ Division of Geriatrics, Department of Family and Community Medicine, Faculty of medicine, The University of Jordan, Amman, Jordan.

^4^ School of Medicine, the University of Jordan, Amman, Jordan.

^5^ Professor of Surgery, Department of General Surgery, Jordan University Hospital, The University of Jordan, Amman, Jordan.

^6^ The Cambridge Center for Sport and Exercise Sciences, Anglia Ruskin University, Cambridge, UK

^7^ Department of Family and Community Medicine, Faculty of medicine, The University of Jordan, Amman, Jordan.

**Corresponding author:**

Mohammad Abufaraj, MD

Division of Urology,

Department of Special Surgery,

The University of Jordan,

11942 Amman, Jordan

Email: [mabufaraj@ju.edu.jo](mailto:mabufaraj@ju.edu.jo)

Telephone: +9625353444-Ext: 2369

**Supplementary Table S1: Jordanians and Syrians Sample Size and PSUs per Governorate.**

| **Governorate** | **Proposed sample from**  **Jordanians** | **Proposed sample from Syrians** | **No. of PSU’s (Blocks)** |
| --- | --- | --- | --- |
| **Amman** | 335 | 336 | **42** |
| **Zarqa** | 191 | 193 | **24** |
| **Irbid** | 216 | 216 | **27** |
| **Mafraq** | 116 | 116 | **15** |
| **Total** | **858** | **860** | **108** |

**Supplementary Table S2: Participants’ response to Katz Index of Independence in Activities of Daily Living (ADLs) items**

|  | **Total** | **Jordanian** | **Syrian** |  |
| --- | --- | --- | --- | --- |
| **ADL items** | **Dependent**  **N (%)** | **Dependent**  **(%)** | **Dependent**  **(%)** | ***P* value** |
| **Bathing** | 408 (23.7) | 202 (23.5) | 206 (23.7) | 0.800 |
| **Dressing** | 326 (18.98) | 167 (19.4) | 159 (18.5) | 0.639 |
| **Toileting** | 352 (20.50) | 173 (20.1) | 179 (20.9) | 0.702 |
| **Transferring** | 418 (24.33) | 214 (24.9) | 204 (23.8) | 0.593 |
| **Continence** | 105 (6.11) | 53 (6.2) | 52 (6.1) | 0.930 |
| **Feeding** | 127 (7.40) | 72 (8.4%) | 55 (6.4) | 0.120 |
| **Dependent in at least one ADL** | 649 (37.75) | 329 (38.3) | 320 (37.3) | 0.682 |
| **Total score (Mean ± Standard deviation)** | 4.99 ± 1.61 | 4.98±1.63 | 5.00±1.60 | 0.720 |
